# Reply to the Letter to the Editor on: impact of screen use on behavior and sleep in patients with autism spectrum disorder

**DOI:** 10.1055/s-0046-1823654

**Published:** 2026-06-23

**Authors:** Matheus Eugênio de Sousa Lima, Lívia Maria Eugênio Lopes, Fernanda Eugênio de Sousa Lima

**Affiliations:** 1 Hospital de Saúde Mental Professor Frota Pinto, Núcleo de Atenção à Infância e Adolescência, Fortaleza CE, Brazil.; 2 Universidade de Fortaleza, Faculdade de Medicina, Fortaleza CE, Brazil.

Dear Editors,

We appreciate the interest in our review and thank the authors for their important comments regarding our work. We believe the relationship between screen use, behavior, and sleep in autism spectrum disorder (ASD) is a very relevant topic in modern neuroscience, and constructive academic dialogue is essential in refining scientific interpretation. We welcome the opportunity to clarify certain methodological decisions and to contextualize our findings within the scope and objectives of our study.

Regarding the bibliographic search strategy, the selection of Medline/PubMed and SciELO was deliberate and aligned with the objectives and the editorial scope of Arquivos de Neuro-Psiquiatria, which emphasize accessibility and reproducibility, particularly in our context of non-profit study. Although additional databases such as Embase or Scopus could potentially broaden the search, previous analyses of database coverage have demonstrated that PubMed captures most of the peer-reviews biomedical literature.

It is important to emphasize that our methodological approach followed an adapted Preferred Reporting Items for Systematic reviews and Meta-Analyses (PRISMA) framework, 1 allowing transparency in the identification, screening, and selection of studies. Meta-analyses were intentionally excluded from the eligibility criteria because the aim of the review was to synthesize primary evidence rather than reproduce previously aggregated analyses. Sources such as the Cochrane Library were not included in the search strategy for this reason.

The terms “screen time” and “smartphone” were selected based on their consistent use in the last decade of research examining media exposure and neurodevelopmental outcomes. We believe that descriptors such as *digital media* or *internet* would not fit in with our strategy because they would likely have increased variability across studies and reduced the clarity of associations.

Qualitative indications related to patterns of digital media use were not presented in the results section either for the same reason, as well as for their inconsistent presence in the available literature. Dimensions such as parental mediation, content type, and contextual use that were reported in the selected studies used different instruments and conceptual frameworks, which is why we choose not to represent them in the results. The review focused on measurable behavioral and sleep-related outcomes. 2

In summary, we thank the letter's authors for their engagement and constructive observations. We believe that our methodological choices remain consistent with the objectives of the study and provide relevant contribution to the current literature addressing the complex relationship between screen exposure and neurodevelopmental outcomes in ASD. As qualitative patterns of engagement, contextual determinants of screen exposure, and potential therapeutic applications of digital technologies were beyond the scope of our review, we believe they represent valuable directions for future research. We, therefore, welcome and encourage further reviews or empirical investigations addressing these perspectives, which may complement the evidence emphasized in our study and contribute to a more comprehensive understanding of the subject.
